# Physicochemical Properties, Stability and Texture of Soybean-Oil-Body-Substituted Low-Fat Mayonnaise: Effects of Thickeners and Storage Temperatures

**DOI:** 10.3390/foods11152201

**Published:** 2022-07-24

**Authors:** Wan Wang, Chuanbing Hu, Hong Sun, Jiale Zhao, Cong Xu, Yue Ma, Jiage Ma, Lianzhou Jiang, Juncai Hou

**Affiliations:** College of Food Science, Northeast Agricultural University, Harbin 150030, China; 13159806631@163.com (W.W.); m18445114013@163.com (C.H.); sun15065560334@163.com (H.S.); zjlneau714@163.com (J.Z.); 15636116265@163.com (C.X.); myue09418@neau.edu.cn (Y.M.); jiage_ma@neau.edu.cn (J.M.); jlzname@neau.edu.cn (L.J.)

**Keywords:** soybean oil body, mayonnaise, thickener, storage stability, texture

## Abstract

With the increasing consumer demand for low-fat and low-cholesterol foods, low-fat mayonnaise prepared from soybean oil body (SOB) substitute for egg yolk has great consumption potential. However, based on previous studies, it was found that the stability and sensory properties of mayonnaise substituted with SOB were affected due to there being less lecithin and SOB containing more water. Therefore, this study investigated the effects of different ratios of xanthan gum, pectin and modified starch as stabilizers on the apparent viscosity, stability, texture and microstructure of SOB-substituted mayonnaise. It was found that the apparent viscosity and stability of SOB-substituted mayonnaise increased significantly when xanthan gum, pectin and modified starch were added in a ratio of 2:1:1. Meanwhile, the emulsified oil droplets of SOB-substituted mayonnaise were similar in size and uniformly dispersed in the emulsion system with different thickener formulations. In addition, the storage stability of SOB-substituted mayonnaise was explored. Compared with full egg yolk mayonnaise, SOB-substituted mayonnaise had better oxidative stability and bacteriostatic, which is important for the storage of mayonnaise. This study provided a theoretical basis for the food industry application of SOB. Meanwhile, this study provided new ideas for the development and storage of low-fat mayonnaise.

## 1. Introduction

Mayonnaise is an oil-in-water emulsion widely used as a traditional condiment for its creamy texture and special flavor. Traditional mayonnaise contains 65–80% fat, which contributes to its good flavor and texture [1]. In a mayonnaise recipe, egg yolks are the most important ingredient for maintaining stability. The lecithin in egg yolk is often used as an emulsifier and has the function of reducing surface tension, emulsion stability and oxidative stability due to the phospholipids and fatty acids in it [2,3].

The global mayonnaise market is mainly driven by a growing demand for convenience food and the increasing utilization of mayonnaise [2]. However, due to the high calorie and cholesterol content of egg yolks, excessive consumption of mayonnaise is often thought to induce health-related problems. At the same time, major problems faced by mayonnaise manufacturers also include fat oxidation during storage, which leads to off-flavors and tastes in the product, as well as reduced nutritional value and food safety [4]. These factors may reduce consumer acceptance of mayonnaise. As consumers demand healthy diets, many researchers have attempted to add plant-based emulsifiers or thickeners to substitute all or part of the egg yolk [1,5]. However, low-fat mayonnaise requires additional ingredients to maintain its stability provided it maintains a texture similar to the original product. In other words, using alternative emulsifiers and fat substitutes may affect the flavor, texture and acceptability of mayonnaise [6].

Therefore, based on consumers′ demands for a healthy diet and avoiding or delaying lipid oxidation, soybean oil body (SOB) was used to substitute for egg yolk to prepare mayonnaise in this study, which could reduce the cholesterol and fat content and increase the level of unsaturated fatty acids in mayonnaise [7]. Meanwhile, SOB contains antioxidant bioactive substances such as tocopherols and phytosterols, which are of great significance to the storage stability of mayonnaise [8,9]. However, it was found that SOB substitute for egg yolk reduced the apparent viscosity and emulsion stability of low-fat mayonnaise, thereby affecting the sensory score of mayonnaise in previous studies. The addition of thickeners can impart long-term emulsion stability by thickening the food system (i.e., reducing motion of the system) and by forming a viscous, ordered network in the continuous phase to prevent oil separation [10]. It has been reported that polymers such as xanthan gum, pectin and modified starch have potential as flocculation inducers in emulsification systems. They increase emulsion stability by creating depleting flocculation, reducing the speed of oil droplet movement in the product [11]. Therefore, the addition of xanthan gum, pectin or modified starch (hydroxypropyl distarch phosphate) to the oil-in-water emulsion system can be considered as a promising method to overcome the limitation of SOB substitution for egg yolks causing the quality degradation of low-fat mayonnaise, which can produce low-fat mayonnaise of similar quality to commercial mayonnaise [4].

This study investigated the effects of different types and proportions of thickeners on the texture, apparent viscosity and stability of low-fat mayonnaise prepared by SOB substitution for egg yolk. Meanwhile, the physicochemical properties of SOB-substituted low-fat mayonnaise and full egg yolk mayonnaise during storage were characterized, and the effect of SOB substitution on the storage stability, texture and color properties of mayonnaise was explored. This study not only improves the stability of SOB-substituted mayonnaise, but also further determines the application value of SOB in mayonnaise, provides a theoretical basis for expanding the application of SOB in emulsion food, and also provides a new way for the development of healthy mayonnaise.

## 2. Materials and Methods

### 2.1. Materials

Soybean was provided by Northeast Agricultural University Soybean Research (Harbin, China). Xanthan gum, pectin and modified starch (hydroxypropyl distarch phosphate from corn) were purchased from Henan Wanbang Chemical Technology Co., Ltd. (Zhengzhou, China). All other chemical reagents were of analytical grade.

### 2.2. Preparation of SOB

The preparation of SOB referred to the method described by Zhou et al. with slight modifications [12]. An appropriate amount of clean soybeans was soaked in ionized water (20%, *w*/*v*) for 12 h. The swollen soybeans were ground by a tissue grinder in ice-cold deionized water, and the filtrate was collected through degreased gauze. Sucrose was added to the filtrate and adjusted to a final concentration of 20% (*w*/*v*). The above mixture was centrifuged (10,000 r/min, 20 min) to collect the upper suspended paste, and the above steps were repeated twice. Then, the paste was resuspended in deionized water and centrifuged (10,000 r/min, 20 min) to obtain SOB. Finally, the prepared SOB was treated at 100 °C for 10 min to inactivate the enzymes.

### 2.3. Preparation of Mayonnaise Samples

A total of 14.4 g egg yolk, 3.6 g SOB, 45 g oil, 10 g caster sugar, 15 g water, 2.4 g salt, 0.1 g mustard oil, 0.1 g citric acid and 0.4 g thickener were mixed and stirred (500 rpm, 1 min). The mixture was homogenized at 100 MPa pressure, filled and sterilized to obtain mayonnaise samples. Among them, in order to explore the influence of thickeners on the quality of mayonnaise, 6 kinds of thickener formulations were added: xanthan gum, pectin, modified starch, Compound 1 (the ratio of xanthan gum, pectin and modified starch is 1:1:2), Compound 2 (the ratio of xanthan gum, pectin and modified starch is 2:1:1), Compound 3 (the ratio of xanthan gum, pectin and modified starch is 1:2:1).

### 2.4. Apparent Viscosity

The method for determining the apparent viscosity of mayonnaise samples was based on the report of Orietta et al. with a slight improvement [13]. The apparent viscosity of mayonnaise samples was determined using a rotational rheometer (MARS40, Thermo, Waltham, MA, USA) under conditions of a 1 mm slit distance, 0.5% strain and shear rates of 0.1 to 100 s^−1^.

### 2.5. Emulsion Stability

The method for the determination of the emulsion stability of mayonnaise samples referred to the report of Jiang et al. with slight modifications [14,15]. A total of 3 g of SOB-substituted mayonnaise sample (M_0_) was heated in a water bath at 60 °C for 1 h, and then centrifuged at 8000 r/min for 10 min. Finally, the fat separated from the lower layer was sucked out, and the mass of the remaining mayonnaise (M_1_) was weighed.

Emulsion stability (%) = 100 × M_1_/M_0_ (1)

### 2.6. Thermal Stability

The method for measuring the thermal stability of mayonnaise samples was improved according to the report of Nikzade et al. [16] A total of 3 g of SOB-substituted mayonnaise (W_0_) was placed in an oven at 80 °C for 30 min, and then centrifuged at 8000 r/min for 10 min. Finally, the fat separated from the lower layer was sucked out, and the mass of the remaining mayonnaise (W_1_) was weighed.

Thermal stability (%) = 100 × W_1_/W_0_ (2)

### 2.7. Texture

The method for determining the texture of mayonnaise samples was according to the report of Liu et al. based on the back extrusion technique [17]. The back extrusion unit of the 35 mm diameter compression disc was used. The mayonnaise samples were filled into cylindrical containers (inner diameter: 50 mm, height: 75 mm) with a depth of 50 mm. One cycle was applied at a constant crosshead velocity of 1 mm/s to a sample depth of 40 mm, and then returned. The texture analyzer (TA-XT Plus, SMATA, Godalming, UK) and Texture Expert for Window version 1.22 software were applied to analyze the values of texture properties of the mayonnaise samples (hardness (g), adhesiveness (g·sec), springiness and cohesiveness).

### 2.8. Microstructure

The method of observing the microstructure of mayonnaise refers to a previous report [7]. A total of 1 mL of mayonnaise sample dilution (10% *w*/*v*) was mixed with 20 μL of Nile red isopropanol solution (0.1%) and 25 μL of Nile blue isopropanol solution (0.1%) and protected from light reactions for 30 min. Ultra-high distraction microscopy (Deltavision OMX SR, GE, Boston, MA, USA) was used to observe the microstructure of the mayonnaise samples.

### 2.9. Total Bacteria Count

Mayonnaise samples were serially diluted 10-fold. Then, 100 μL of the dilution was added to the plate count agar medium by pouring. The medium was incubated at 37 °C for 48 h, followed by bacterial counts.

### 2.10. Peroxide Value (PV)

The PV of mayonnaise samples was slightly modified according to the method of Li et al. [18]. A total of 0.15 g of mayonnaise sample was mixed with 1.5 mL of isooctane/isopropanol mixture (2:1, *v*/*v*) and centrifuged (2000 r/min, 5 min) to collect the supernatant. A total of 0.5 mL supernatant with 20 μL KSCN (3.94 mol/L), 20 μL FeSO_4_ (0.072 mol/L), 3 mL methanol/n-butanol mixture (2:1, *v*/*v)* was mixed and reacted in the dark for 20 min. The absorbance of the reaction solution was measured at a wavelength of 510 nm. The PV of the mayonnaise samples on days 0, 5, 10, 15 and 20 was measured at different storage temperatures.

### 2.11. Thiobarbituric Acid Reactive Substance (TBARS)

The method for determining the TBARS of mayonnaise samples was as described by Li et al. with modifications [18]. A total of 0.3 g of mayonnaise sample, 1 mL of trichloroacetic acid solution (10%) and 2.5 mL of thiobarbituric acid solution (1%) were mixed and boiled for 30 min. The mixture was mixed with 0.5 mL of chloroform, shaken evenly and centrifuged (6000 r/min, 15 min) to collect the supernatant. The absorbance of the supernatant was measured at a wavelength of 532 nm. The TBARS of mayonnaise samples on days 0, 5, 10, 15 and 20 was measured at different storage temperatures.

### 2.12. PH

The method for the determination of the mayonnaise samples was as described by Rujirat et al., with modifications [19]. A total of 2 g of mayonnaise and 18 mL of water were mixed and dispersed evenly with a vortex shaker, and then the pH of the mixture was measured with a pH meter (PB-10, Sartorius, Gottingen, Germany).

### 2.13. Color Properties

The determination of the color properties of the mayonnaise samples was described by Wang et al. [7]. The light-dark value (L*), red-green value (a*), and yellow-blue value (b*) of mayonnaise samples were measured by a colorimeter (ZE6000, Nippon Denshoku, Tokyo, Japan).

### 2.14. Statistical Analysis

All data were expressed as means ± standard deviation. Data were analyzed by one-way ANOVA with Tukey′s correction for multiple comparisons. The figures were drawn by Prism 8.0 software (GraphPad, La Jolla, CA, USA).

## 3. Results

### 3.1. Apparent Viscosity of SOB-Substituted Mayonnaise

**Figure 1:**
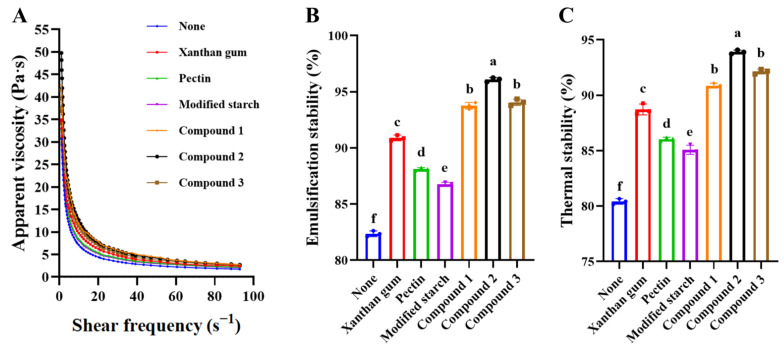
Effects of different stabilizers on the (**A**) apparent viscosity, (**B**) emulsification stability and (**C**) thermal stability of SOB-substituted mayonnaise. Compound 1 represented xanthan gum, pectin and modified starch in a ratio of 1:1:2, compound 2 represented xanthan gum, pectin and modified starch in a ratio of 2:1:1, and compound 3 represented xanthan gum, pectin and modified starch in a ratio of 1:2:1. Different lowercase letters indicated significant difference (*p* < 0.05).

The apparent viscosity of SOB-substituted mayonnaise is shown in Figure 1A. As the shear frequency increased in the range of 0.1–100 s^−1^, the apparent viscosity of SOB-substituted mayonnaise gradually decreased, resulting in shear thinning. This is because the aggregated droplets and hydrocolloid networks were disrupted by shear forces, thus exhibiting a lower apparent viscosity [20].

The addition of thickener increased the apparent viscosity of SOB-substituted mayonnaise, and the thickening effect of xanthan gum was higher than that of pectin and modified starch. This may be because xanthan gum could combine with water molecules, thereby reducing the content of free water in mayonnaise to improve the gel strength of SOB-substituted mayonnaise, resulting in a complex gel matrix with viscoelastic properties [21]. Meanwhile, the results of this study are consistent with those of Karen-Gisseth et al. Compared with pectin and starch, xanthan gum enables a stronger network structure, interacting units, and a high consistency index to be observed in hydrocolloids, which is attributed to the rigidity, rod-like conformation and high molecular weight of xanthan gum [22]. In addition, the compounded thickener has a stronger thickening effect on SOB-substituted mayonnaise. When the compound ratio of xanthan gum, pectin and modified starch was 2:1:1, the apparent viscosity of SOB-substituted mayonnaise was the highest. This may be due to the fact that compounding of the thickener reduced the fluidity of the oil and made the three-dimensional network oil-in-water structure of the SOB-substituted mayonnaise more compact, thereby enhancing the apparent viscosity [17]. This result is consistent with the findings of Laleh et al., that the thickening effect of nanofibrous cellulose-complexed guar pectin resulted in a higher apparent viscosity and stability of the samples, with no significant difference in mouthfeel from the commercial control samples [23].

### 3.2. Emulsion Stability and Thermal Stability of SOB-Substituted Mayonnaise

A previous study by our group found that SOB-substituted mayonnaise contained more water, so a thickener needs to be added to improve the compactness of the mayonnaise to improve the stability [24]. The effects of adding thickeners on the emulsion stability and thermal stability of SOB-substituted mayonnaise are shown in Figure 1B,C. The addition of thickeners could significantly improve the emulsion stability and thermal stability of SOB-substituted mayonnaise (*p* < 0.05). Among them, the effect of adding xanthan gum on its emulsion stability and thermal stability was significantly higher than that of adding pectin and modified starch. In addition, compared with adding a single thickener, the combination of thickeners can improve the stability of SOB-substituted mayonnaise. When the compound ratio of xanthan gum: pectin: modified starch was 2:1:1, the emulsion stability of SOB-substituted mayonnaise was (97.19 ± 1.12)%, and the thermal stability was (95.59 ± 0.98)%, which was significantly higher than other formulations (*p* < 0.05). This is because xanthan gum was able to help stabilize droplets against coalescence through a variety of intermolecular interactions, including electrostatics, polymer steric interactions, hydrogen bonding, hydrophobic association and cation-mediated cross-linking [25]. Meanwhile, the modified starch acted as a filler in the emulsion, and the pectin acted as a network in the emulsion [10]. Xanthan gum, pectin and modified starch exerted their respective functional properties and formed a three-dimensional network conformation through synergistic action, thereby enhancing the emulsion stability and thermal stability of SOB-substituted mayonnaise [26]. Furthermore, the apparent viscosity of the emulsion is believed to be positively correlated with stability. The addition of thickener increased the apparent viscosity of mayonnaise, which indicated that the steric hindrance and electrostatic repulsion between droplets gradually increased, thus improving the stability of mayonnaise emulsion [27].

### 3.3. Texture of SOB-Substituted Mayonnaise

The effect of the thickener on the texture of SOB-substituted mayonnaise is shown in Table 1. Hardness refers to the force required to compress the food between the molars, and adhesiveness refers to the force required to overcome the attraction between the mayonnaise and the surface of other materials [28]. There was no significant difference in the springiness and cohesiveness of SOB-substituted mayonnaise with the thickener (*p* > 0.05), but the thickener could significantly improve the hardness and adhesiveness of SOB-substituted mayonnaise (*p* < 0.05). This is because the thickener could absorb the oil and water and reduce the fluidity of the SOB-substituted mayonnaise, which made the oil-in-water structure more compact and the texture more uniform, thereby increasing the hardness and adhesiveness of the SOB-substituted mayonnaise [29]. In addition, compared with adding a single thickener, the combination of thickeners could significantly improve the hardness and adhesiveness of SOB-substituted mayonnaise. When the compound ratio of xanthan gum: pectin: modified starch was 2:1:1, the hardness and adhesiveness of SOB-substituted mayonnaise were significantly higher than those of other recipes (*p* < 0.05), which was consistent with the trend in apparent viscosity. According to Nikzade et al., apparent viscosity could partly determine the texture of mayonnaise, and hardness and adhesiveness can be affected by apparent viscosity [16].

### 3.4. Microstructure of SOB-Substituted Mayonnaise

**Figure 2:**
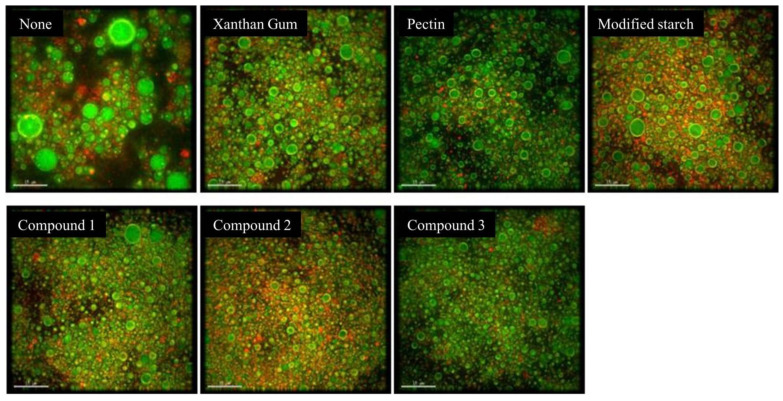
Effects of different stabilizers on the microstructure of SOB-substituted mayonnaise. Compound 1 represented xanthan gum, pectin and modified starch in a ratio of 1:1:2, compound 2 represented xanthan gum, pectin and modified starch in a ratio of 2:1:1, and compound 3 represented xanthan gum, pectin and modified starch in a ratio of 1:2:1.

The microstructure of mayonnaise is related to the texture and viscoelasticity, which in turn determines the quality and acceptability of the product [30]. The effect of thickeners on the microstructure of SOB-substituted mayonnaise was analyzed by ultra-high distraction microscopy, as shown in Figure 2. SOB-substituted mayonnaise without added thickener had a larger droplet size, less aggregation, and uneven droplet distribution. The addition of xanthan gum, pectin or modified starch resulted in the SOB-substituted mayonnaise emulsion system containing smaller-sized and well-distributed droplets, indicating improved stability. In addition, the combination of thickeners resulted in smaller and more aggregated droplet sizes of SOB-substituted mayonnaise, which improved the stability of SOB-substituted mayonnaise compared with single thickeners. Therein, when the compounding ratio of xanthan gum, pectin and modified starch was 2:1:1, the droplets were almost the same in size and uniformly dispersed in the emulsion matrix. Moreover, from the perspective of the microstructure, there was no obvious oil droplet coalescence, which meant that the stability was the best [21]. This may be because the compound thickener makes the SOB-substituted mayonnaise form a tight space network structure to distribute the droplets evenly, thereby improving the quality of SOB-substituted mayonnaise [31]. In addition, the close packing of droplets facilitates the interaction between droplets and is the main reason for the structural stability of mayonnaise [30]. This result is consistent with the texture and stability results of thickeners in SOB-substituted mayonnaise.

### 3.5. Total Bacteria Counts in Mayonnaise during Storage

**Figure 3:**
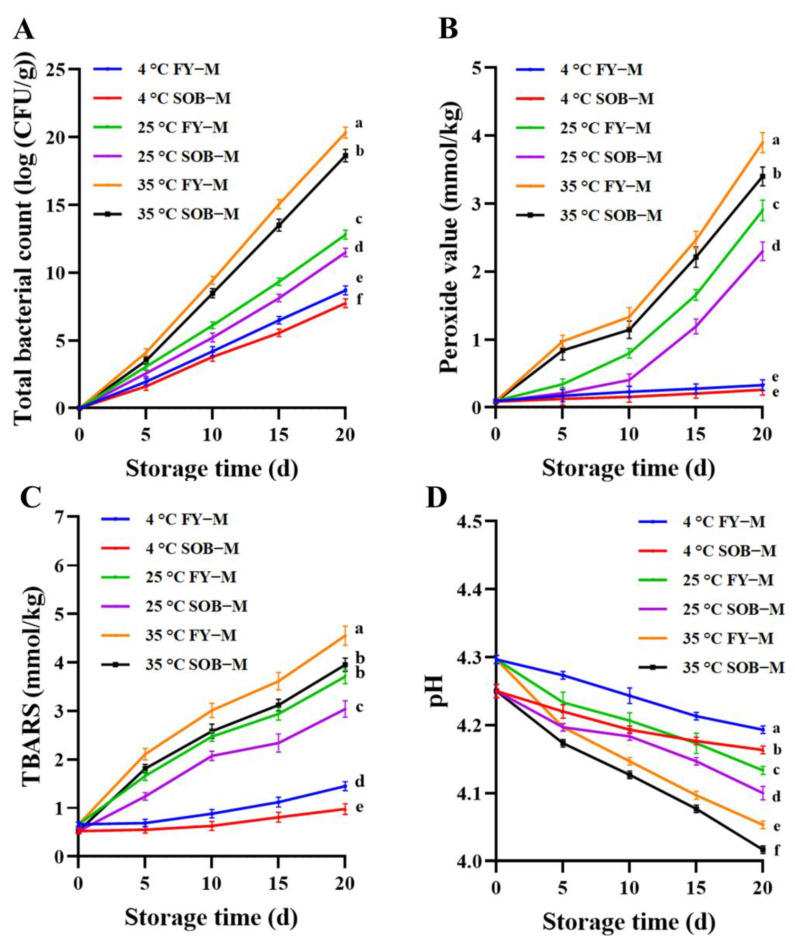
The (**A**) total bacteria count, (**B**) peroxide value, (**C**) thiobarbituric acid reactive substance, and (**D**) pH of full egg yolk mayonnaise (FY−M) and SOB-substituted mayonnaise (SOB−M) during storage. Different lowercase letters indicated significant difference (*p* < 0.05).

Bacteria are generally closely related to human life, but the proliferation of bacteria can lead to food spoilage and affect food safety [32]. The total bacterial count of mayonnaise during storage is shown in Figure 3A. After 20 days of storage at different temperatures, the total bacteria count in mayonnaise increased significantly (*p* < 0.05). The total bacterial count of full egg yolk mayonnaise was 8.69 log(CFU/g), 12.8 log(CFU/g) and 20.35 log(CFU/g), and the total bacterial count of SOB-substituted mayonnaise was 7.74 log(CFU/g), 11.5 log(CFU/g) and 18.65 log(CFU/g) at the storage temperature of 4 °C, 25 °C and 35 °C, respectively. With the increase in storage temperature, the total bacterial count increased faster because low temperatures inhibited the growth activity of bacteria [33]. However, when the optimal growth temperature of bacteria is reached, the bacteria will multiply rapidly, thus affecting the quality of mayonnaise [34,35]. In addition, the total bacterial count of SOB-substituted mayonnaise was consistently lower than that of whole-egg mayonnaise during storage. This may be due to the presence of bacteriostatic bioactive substances in SOB, such as tocopherols, phytosterols, etc. These bacteriostatic bioactive substances are natural preservatives that can inhibit the growth of bacteria in food, which is of great significance for prolonging the shelf life of food and improving oxidative stability [7,36].

### 3.6. Oxidative Stability of Mayonnaise during Storage

The key reason for the destabilization of mayonnaise may be the oxidation of fatty acids. Lipid oxidation can result in toxic substances, unpleasant tastes and odors that can reduce the shelf life, safety and consumer acceptance of commercial mayonnaise [37]. The storage oxidative stability of SOB-substituted mayonnaise and whole mayonnaise stored under different temperature conditions (4 °C, 25 °C, 35 °C) was investigated in Figure 3B,C. The peroxide value (PV) and thiobarbituric acid reactants (TBARS) of all mayonnaise samples increased significantly with storage time and storage temperature (*p* < 0.05), indicating that lipid oxidation occurred during the storage of mayonnaise, and the higher the temperature, the faster the lipid oxidation rate. The results for the oxidative stability of mayonnaise were consistent with the experimental results of Fisk et al., which may be due to the fact that higher storage temperatures could increase the activity of lipase and accelerate the oxidation rate of lipids [38]. In addition, the PV and TBARS of SOB-substituted mayonnaise were lower than those of full egg yolk mayonnaise at the same storage temperature, indicating that SOB substitution in mayonnaise could improve its oxidative stability. This may be because SOB contained tocopherols, isoflavones and other antioxidant bioactive components, which improved the storage stability of mayonnaise and slowed down the oxidation rate of lipids [39].

### 3.7. pH of Mayonnaise during Storage

Mayonnaise is a relatively acidic product. Furthermore, pH is the main parameter that affects the shelf life and consumer acceptance of mayonnaise [40]. The pH values of mayonnaise stored at different temperatures for 20 d are shown in Figure 3D. The pH of all mayonnaise samples decreased significantly with an increasing storage time and storage temperature (*p* < 0.05). This may be due to lipid oxidation that occurred during the storage of mayonnaise, which released fatty acids, which lower the pH of mayonnaise. In addition, double bond oxidation of unsaturated fatty acids, hydrolysis of fats and the release of free fatty acids all increased the acidity of mayonnaise during storage, resulting in a decrease in pH [41]. Since SOB contains tocopherols, isoflavones and other antioxidant bioactive components, this inhibits lipid oxidation, thereby delaying the pH reduction of SOB-substituted mayonnaise [42].

### 3.8. Hardness and Adhesiveness of Mayonnaise during Storage

**Figure 4:**
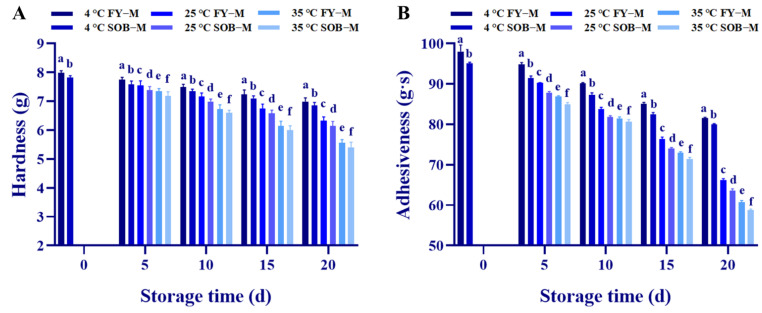
Effects of different stabilizers on (**A**) hardness, (**B**) adhesiveness of full egg yolk mayonnaise (FY−M) and SOB-substituted mayonnaise (SOB−M) during storage. Different lowercase letters indicated a significant difference (*p* < 0.05).

The hardness and adhesiveness of mayonnaise are shown in Figure 4A,B, showing mayonnaise stored under different temperature conditions (4 °C, 25 °C, 35 °C) for 20 d. The hardness and adhesiveness of the mayonnaise decreased significantly with an increasing storage time (*p* < 0.05). Meanwhile, under the same storage time, the higher the storage temperature, the faster the reduction rate of the hardness and adhesiveness of mayonnaise. This may be due to the fact that the oil-in-water emulsion system of mayonnaise is destroyed by lipid oxidation during storage, and the interaction between droplets disappears, resulting in a decrease in the hardness and adhesiveness of mayonnaise, which is a phenomenon of thinning [43]. In addition, SOB-substituted mayonnaise was consistently lower in hardness and adhesiveness than full egg yolk mayonnaise for the same storage time and storage temperature. This is because SOB substitution for egg yolk increased the moisture content and decreased the fat content of mayonnaise, resulting in a decrease in apparent viscosity, which affected the initial hardness and adhesiveness of SOB-substituted mayonnaise [17]. However, SOB-substituted mayonnaise decreased hardness and adhesiveness at a lower rate than full egg yolk mayonnaise with an increasing storage time. This is because the antioxidants active in SOB-substituted mayonnaise could prevent it from losing hardness and adhesiveness during storage by inhibiting bacterial growth and pH value reduction [44].

### 3.9. Color Properties of Mayonnaise during Storage

**Figure 5:**
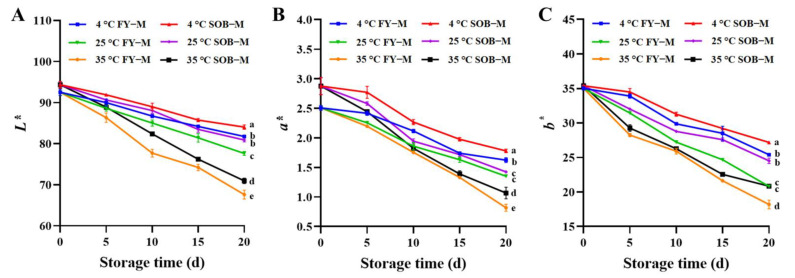
The (**A**) light-dark value, (**B**) red-green value, and (**C**) yellow-blue value of full egg yolk mayonnaise (FY−M) and SOB-substituted mayonnaise (SOB−M) during storage. Different lowercase letters indicated a significant difference (*p* < 0.05).

Mayonnaise is a high-fat food that is prone to oxidative deterioration due to the auto-oxidation of unsaturated fatty acids, which affects color properties [30]. The color properties of mayonnaise are shown in Figure 5, showing mayonnaise stored under different temperature conditions (4 °C, 25 °C, 35 °C) for 20 d. The light-dark value (L*), red-green value (a*) and yellow-blue value (b*) of mayonnaise were determined to evaluate color properties. The L*, a* and b* values of all mayonnaise samples showed a decreasing trend during storage, and the higher the storage temperature, the faster the decreasing rate. This may be due to lipid oxidation during storage, darkening the color of the mayonnaise [43]. In addition, the total bacterial count and lipase activity of mayonnaise were significantly increased due to the elevated storage temperature, resulting in aggravated lipid oxidation and thus affecting the texture of mayonnaise. Changes in the texture of mayonnaise affect the reflection of light, thereby reducing the a* and b* values [38]. In addition, the color properties of SOB-substituted mayonnaise were consistently lower than those of full egg yolk mayonnaise at the same storage time and storage temperature because SOB was white but the egg yolk was dark yellow. In addition, SOB substitution for egg yolks increased the moisture content of the mayonnaise, causing the color to be diluted [45].

## 4. Conclusions

In this study, thickeners were able to enhance the apparent viscosity, emulsion stability and thermal stability of SOB-substituted mayonnaise, while improving its hardness and adhesiveness. When the compounding ratio of xanthan gum: pectin: modified starch was 2:1:1, mayonnaise had the highest stability and excellent texture properties, which may be related to their respective functional properties allowing for the formation of a three-dimensional network conformation in SOB-substituted mayonnaise through synergy. In addition, SOB-substituted egg yolks could delay the lipid oxidation of mayonnaise and improve the oxidative stability of SOB-substituted mayonnaise during storage, compared with the full egg yolks mayonnaise. This study provided new insight for the food industry’s application of SOB and the development and storage of low-fat mayonnaise.

## Figures and Tables

**Table 1:** Effect of stabilizer formulation on texture of SOB-substituted mayonnaise.

Stabilizer	Hardness (g)	Adhesiveness (g·sec)	Springiness	Cohesiveness
^1^ none	^3^ 6.56 ± 0.16 ^f^	68.52 ± 0.59 ^f^	0.41 ± 0.04 ^a^	0.32 ± 0.03 ^a^
xanthan gum	7.25 ± 0.08 ^c^	73.36 ± 0.57 ^c^	0.43 ± 0.04 ^a^	0.33 ± 0.04 ^a^
pectin	7.02 ± 0.13 ^d^	71.89 ± 0.54 ^d^	0.39 ± 0.06 ^a^	0.34 ± 0.02 ^a^
modified starch	6.97 ± 0.15 ^e^	71.60 ± 0.48 ^e^	0.44 ± 0.03 ^a^	0.35 ± 0.02 ^a^
^2^ compound 1	7.82 ± 0.06 ^b^	76.97 ± 0.22 ^b^	0.41 ± 0.02 ^a^	0.34 ± 0.02 ^a^
compound 2	8.41 ± 0.13 ^a^	79.53 ± 0.47 ^a^	0.42 ± 0.05 ^a^	0.32 ± 0.03 ^a^
compound 3	7.71 ± 0.12 ^b^	75.13 ± 0.34 ^b^	0.40 ± 0.03 ^a^	0.34 ± 0.04 ^a^

^1^ None represented no thickeners were added. ^2^ Compound 1 represented xanthan gum, pectin and modified starch in a ratio of 1:1:2; compound 2 represented xanthan gum, pectin and modified starch in a ratio of 2:1:1; compound 3 represented xanthan gum, pectin and modified starch in a ratio of 1:2:1. ^3^ Different lowercase letters in the same column indicated significant differences (*p* < 0.05).

## Data Availability

Data are contained within the article.
